# Supplementary material for: The effects of a synthetic epidermis spray on secondary intention wound healing in adult horses
Source: PLoS One. 2024 Mar 7;19(3):e0299990. doi: 10.1371/journal.pone.0299990 (PMC10919598; doi:10.1371/journal.pone.0299990)
Supplement: Attachment — Submitted filename: Response to Reviewers.docx [file pone.0299990.s002.docx]

Response to Reviewers,

Reviewer #1 :

- Comment: Lines 83-85 This step introduces extremely high variability in the wound environment. Was there any type of microbiological understanding (ie. Culture) of the wound environment prior to treatment?

Answer: No there wasn’t. We believe that the environment contained multiple bacteria as it is on the field. We did not focus on the infection risk as it is rarely a problem in superficial wounds on horse’s limb. Culture is usually not performed on superficial wounds in clinical cases.

- Comment: Lines 126-127 Area, height, depth and volume are related measurements and should be treated as such statistically. Also, Can you explain the difference between the measurements: depth and height? Figure 6 should detail this but seems to only be an illustration of the software interface.

Answer: The wounds were analysed for area, volume, height and depth because wound healing on horse’s limb appears irregular with the granulation tissue being more prominent on a side. The height and depth values are giving information about the “landforms” of the granulation tissue. For example: the higher height would be where the granulation tissue was the more prominent and depth where there was poor granulation tissue.

- Comment: Lines 136 “The numbers of knots used, respectively 8, 5, 8 and 6”, please explain this part of the text. I cannot relate to this.

Answer: This part of the text was adapted accordingly.

Reviewer #2:

- Comment: Usually, in-text reference numbers should be inserted to the left or inside colons/semi-colons and placed outside or after full stops and commas. Please apply to the entire document.

Answer: I am not sure to understand this comment. I followed the journal requirement concerning the reference https://journals.plos.org/plosone/s/file?id=wjVg/PLOSOne_formatting_sample_main_body.pdf

- L.49-53 I usually place the hypothesis before your objective as it describes how you plan to challenge your hypothesis, but it is up to debate...

Answer: we followed your advice

- Comment: L.60 How do you justify the small number of horses in your study?

Answer: Above all, it's to respect the 3Rs (Replace, Reduce, and Refine) of animal research considered a systematic approach to animal experimentation that puts the well-being of the animal’s in front and by the fact that the experimental model we have used has already been validated many times in this large, expensive animal.

- Comment: L.71 Could you comment on the reason why you did not clip the hair on the same day as the surgery? As for our veterinary patients, clipping is recommended immediately before surgery.

Answer: To facilitate the ease of the next day. It is true however that we should have done it the same day if we want to mimic natural occurring trauma.

- Comment: L.75 Could you comment on the reason why you elected not to perform a surgical scrub before surgery? I assume it was to try to mimic a traumatic injury and/or to avoid skin flora disruption...it may be worth mentioning it.

Answer: indeed, we wanted to be as close as possible of the reality for natural occurring wound. We will add an explanation in the manuscript.

- Comment: L.102 How did you standardize the distance of UV exposition?

Answer: Subjectively, it was the width of the manipulator's open hand (about 30 cm). A distance that could vary by a few centimetres if the horse moved its opposite limb.

- Comment: L.103-106 Why is your control to bandage wounds? Why not let the wounds heal without any bandages? You need to justify it or at least discuss it later on. As you mentioned in your discussion, bandage use promotes EGT, so this complication is to be expected down the line, so you need to justify your choice of control treatment. Otherwise, it will be difficult to compare the prevalence of EGT in both of your groups because if the wounds treated with the spray did not develop EGT, is that because they were not bandaged? Would naked wounds have developed EGT?

Answer: we believe that another study should compare the product to naked wounds. However, in this study, we chose to compare to standard bandaging technique as most of the clinician cover wounds using this technique when facing superficial wounds. We explain this more clearly in the text.

- Comment: L.107 Why 4 days? Knowing what is reported in the literature about EGT and bandages, would a more frequent bandage change have been more appropriate to avoid EGT development?

Answer: We chose to change it every 4 days as we believe veterinarian on the fields commonly change bandages every 3 to 5 days.

- Comment: L.115 What were your criteria for each category of your contraction and epithelialization grading system? I understand it is a subjective grading system, but how could you visually assess contraction or epithelialization properly to classify it as mild, moderate, or severe?

Answer: As it is always the same assessor who inspects the wounds, he could more easily assess whether a smooth whitish-pink edge was developing around the periphery (epithelialization) and, or whether a reduction in the granulation surface was observed (contraction).

- Comment: L.162-163 It would be interesting to know how long it took for the wounds to heal completely between both groups.

Answer: Regarding the area, only 16 wounds out of 46 achieve a complete healing (defined by a null wound area) during the follow-up. This is why we compared the wound kinetics instead of the time to complete healing.

- Comment: L164-199 P-values should be mentioned in the text for significant results

Answer: P-values were added in the text.

- Comment: L.191 Did you compare statistically treatments between locations? I do not see any p-value related to that

Answer: We added the detail about the comparison between locations.
