# Supplementary material for: The effects of a synthetic epidermis spray on secondary intention wound healing in adult horses
Source: PLoS One. 2024 Mar 7;19(3):e0299990. doi: 10.1371/journal.pone.0299990 (PMC10919598; doi:10.1371/journal.pone.0299990)
Supplement: Attachment — Submitted filename: Response to Reviewers.docx [file pone.0299990.s003.docx]

Response to Reviewer 2,

Comment: L.115 What were your criteria for each category of your contraction and epithelialization grading system? I understand it is a subjective grading system, but how could you visually assess contraction or epithelialization properly to classify it as mild, moderate, or severe?
Answer: As it is always the same assessor who inspects the wounds, he could more easily assess whether a smooth whitish- pink edge was developing around the periphery (epithelialization) and, or whether a reduction in the granulation surface was observed (contraction).''

Reviewer answer: That does not answer the question. For example, what is the difference between mild epithelialization, moderate epithelialization and severe epithelization? Same question for contraction. You used a subjective grading system without a reference atlas or measurements describing each category. That questions the pertinence of such assessment and its value in the paper. Later in the results section, you even mention that these assessments are not analyzed, so why do you bother explaining this subjective assessment? You could just mention that wounds were evaluated at each time point for the presence or absence of epithelization, contraction, EGT, etc.

Answer: please find a table below with the criteria for the grading system. However, as it was not analyzed, we will follow what you suggested.

| Criteria/Grade | Absent (1) | Mild (2) | Moderate (3) | Severe (4) |
| --- | --- | --- | --- | --- |
| Secretion | 0 | Humid on surface | Flows slightly beyond wound margin | Flows beyond wound margin |
| Granulation tissue | 0 | Under skin level | At skin level | Exceeds skin level |
| Epithelialization | 0 | Border of epithelium around entire periphery | Band of epithelium around entire periphery | Band of epithelium >0.5cm |
| Contraction | 0 | Wound surface reduced with a band of epithelium >0.5cm | Wound surface reduced with a band of epithelium <0.5cm | Wound reduced surface with a border of epithelium |

Comment: L269 I already made a comment on the number of horses used. This has not be addressed. If you mention that one of your limitations is ‘the small number of horses’ you need to justify it by referring to other work/studies published with significant data with a similar model.

Answer: this protocol was used in a previous article (ref 15) and showed that they could see a statistical difference with 6 horses. We added it on the text.
