# Supplementary material for: The effects of a synthetic epidermis spray on secondary intention wound healing in adult horses
Source: PLoS One. 2024 Mar 7;19(3):e0299990. doi: 10.1371/journal.pone.0299990 (PMC10919598; doi:10.1371/journal.pone.0299990)
Supplement: Attachment — Submitted filename: Response to Reviewers.docx [file pone.0299990.s004.docx]

Response to Reviewer #3:

Comment: The authors set out to evaluate the secondary intention wound healing in the horse's limbs when treated with the synthetic epidermis spray or with a standard bandaging technique. The design and analysis appears to be simple using the generalized least square models with area, volume, height and depth as the dependent variables and time (in days), treatment (T1 vs T2), body part (cannon vs fetlock) and all interactions as the independent variables. The interpretation, as noted below, is not simple. Also the investigators claim that six horses are adequate for the sample size as noted from previous publications. However, the sample size is not discussed and certainly not justified with respect to any of the variables in this presentation.

Simply stated the overall result is that whether the wounds were treated with synthetic epidermis spray or standard bandaging, the time for healing was the same. This is not obvious in light of the time and location effect. The presentation of the results are seen primarily in Table 2. It appears like a multivariate issue with the major variables of interest being time, treatment and location. There are no gross p-values where the authors state in the overall experiment whether there is an independent time, treatment or location effect adjusted for the others. The attempt at explaining the interactions may be reasonable. Table 2 is a summary univariate analysis for the dependent variables in five tables. The organization of the analysis is confusing. There are many analyses and many p-values, resulting in possible multiple comparisons and no adjustment for such.

Also, the unit of analysis is the wound (it appears). The horse may also be a unit of analysis which gets into the issue of intraclass correlation of the treatments across these units. The variance of the estimates could be inflated in this situation.

The unit of analysis issue and multiple tests could possibly be addressed by the authors already as seen on line 157 in which they state that the model therefore included an auto-regressive (first-order) correlation structure that allowed for this or is this addressing the spatial (location) and time correlation? All of this has to be better clarified by the investigators.

Answer: Thank you for your remarks.

1. We clarified the generalized least square model concerning the auto-regressive correlation structure.
2. We included an additional table presenting a simplified version of the analysis, which can be considered a sensitivity analysis, with the horse as the unit of analysis. We computed and averaged the area under the curve for each wound kinetics, grouped by horse and treatment (refer to the table below). The conclusions mirror those of the complex model: the height is observed to decrease with SES bandaging, while the area and volume remain unaffected.

Correction for multiple comparisons does not seem necessary, as observations on the various outcomes at different times are highly dependent.

Concerning the sample size, it was predefined based on material constraints. A posteriori, this sample size proved adequate to highlight the treatment effect on wound height evolution and convincingly demonstrate that the healing duration is not strongly influenced by the type of bandage.

|  |  | **Area under the curve** | | |  | 95% CI | |  |
| --- | --- | --- | --- | --- | --- | --- | --- | --- |
| **Horse** | **Outcome** | **B** | **N** | **Difference** | **Mean** | **lower limit** | **upper limit** | **P** |
| A | Area | 177 | 234 | -57 | 8 | -45 | 61 | 0,70 |
| C |  | 166 | 202 | -36 |  |  |  |  |
| H |  | 172 | 141 | 31 |  |  |  |  |
| I |  | 218 | 172 | 46 |  |  |  |  |
| V |  | 183 | 191 | -8 |  |  |  |  |
| Y |  | 233 | 158 | 75 |  |  |  |  |
| A | Depth | 1,9 | 2,0 | -0,1 | -0,3 | -0,6 | 0,0 | 0,07 |
| C |  | 1,4 | 1,9 | -0,5 |  |  |  |  |
| H |  | 1,2 | 1,9 | -0,7 |  |  |  |  |
| I |  | 1,6 | 1,6 | 0,0 |  |  |  |  |
| V |  | 1,3 | 1,5 | -0,2 |  |  |  |  |
| Y |  | 2,2 | 2,3 | -0,1 |  |  |  |  |
| A | Height | 49 | 51 | -2 | 16 | 1 | 31 | 0,04 |
| C |  | 48 | 42 | 6 |  |  |  |  |
| H |  | 44 | 24 | 20 |  |  |  |  |
| I |  | 55 | 29 | 26 |  |  |  |  |
| V |  | 50 | 40 | 11 |  |  |  |  |
| Y |  | 60 | 24 | 37 |  |  |  |  |
| A | Volume | 16 | 21 | -5 | 8 | -3 | 19 | 0,13 |
| C |  | 17 | 17 | 0 |  |  |  |  |
| H |  | 17 | 7 | 10 |  |  |  |  |
| I |  | 27 | 13 | 14 |  |  |  |  |
| V |  | 19 | 16 | 3 |  |  |  |  |
| Y |  | 31 | 7 | 24 |  |  |  |  |
