# Supplementary material for: The effects of a synthetic epidermis spray on secondary intention wound healing in adult horses
Source: PLoS One. 2024 Mar 7;19(3):e0299990. doi: 10.1371/journal.pone.0299990 (PMC10919598; doi:10.1371/journal.pone.0299990)
Supplement: Attachment — Submitted filename: Response to Reviewers.docx [file pone.0299990.s005.docx]

Response to Reviewer #3:

Comment: The limitations are listed. However, given the small sample size, the authors should put a qualification in the conclusions that, "All statistical inference (p-values especially) must be interpreted with caution, given the size of the sample.”

Answer: Thank you for your remarks. We added the sentence to the manuscript.
